# Address to the Graduates of the Baltimore College of Dental Surgery

**Published:** 1879-01

**Authors:** Julius E. Grammer

**Affiliations:** Rector of St. Peter's P. E. Church, Baltimore, Md.


### ARTICLE II.

### *Address to the Graduates of the Baltimore College of Dental Surgery, March, 5th.,* 1878.

BY REV. JULIUS E. GRAMMER, D. D.

Rector of St. Peter's P. E. Church, Baltimore, Md.

Gentlemen of the Graduating Class.—In completing your curriculum, as students of this time-honored Institution, I congratulate you that you are about to go forth with the credentials of such an honorable career. The college itself will reflect dignity and character upon your profession.

Being the oldest, and, at one time, the only Dental College in the world, we may safely say that it is not only the mother, but the Queen of Dental Colleges. When I think of the mighty roll of over seven hundred graduates which grace its history, and recall the fact, that they have come from every part of this land ; from Canada and the West Indies; from South America and Europe; while they are practising their profession in London, in Berlin, in Paris and Madrid, in Barcelona, in Florence and Rome, in Vienna and St. Petersburg, I may *well say*, "*Quae regio in terris nostri non plena laboris?*" ; and not only so, but when you recall the fact that without exception all the great dentists in Europe are graduates of this school, you may well rejoice that you are to begin under the auspices of a school whose fame has gone into all lands.

It involves a high honor as well as a corresponding responsibility to bear the testimonial of such an institution. The names of Hayden, Harris, Bond and Baxley, among the founders, and of those who have since nurtured and are now guiding the college are an incentive to you to act well your part in the future. Gentlemen, I hail your class to-day as another addition to the long line of witnesses to the advantages of a scientific and high culture in professional pursuits. I see that your curriculum encourages no hope for the superficialist and the pretender. To deserve and earn one of these diplomas, it is necessary to have undergone a training in Surgery, Anatomy, Physiology, Pathology, Therapeutics, Materia Medica, Chemistry, Dental Mechanism and Metallurgy, which has won the approval of critical examiners and given pledge of your aptness for your art. It is a cause for gratulation that such an institution has been founded, throwing a guard around the community to protect it from cruel impostors, and adding another niche in the temple of learning for a profession which is the hand-maid of health, and the nurse of the beautiful and good in our physical and social life. Who can describe the potentiality for good or evil in this delicate and skillful art?

If the face of many is their fortune, you have it in your power to heighten or destroy the prospects of many a fair daughter of Eve, and many a brave son of Adam. If the disposition is said to depend upon the digestion, then who can do more to gladden with cheerful health or to embitter with dyspeptic melancholy the social circle, than the dentist?

If it be allowed that of all the ills that flesh is heir to, there is none more acute and intense than those which arise from disorders and disease in the members which you cure, surely we must hail a good dentist as entitled to rank with St. Luke, the Evangelist, as "the beloved physician." And if I were asked to name one of the most serious offenders against the social conscience and as worthy to be banished from the Republic of Letters, I should name the man who dares to tamper with those delicate organs which affect our health, our speech, our happiness and our comfort as much as any part of our bodily organism.

Probably there is no department of the mechanics of medicine which boasts of greater progress than dentistry. When we compare the improvement in your science, the invention of chairs and instruments, of every appliance for assuaging pain, of subduing and mastering the difficulties of mechanical obstructions; of supplying the wastes in the dynamical economy of physical life; of supplementing the losses caused by disease and age and giving the appearance and the feeling of youth, to those afflicted with premature decline, I may well rank your profession as among the foremost fruits of our modern civilization. There is a poetry and a religion in the silent and effective agencies by which the patient is lulled to unconscious sleep, during which triumphs of dental surgery are won, without the cost of pain, which otherwise must have been lost to science. For there are some operations, gentlemen, in which no success could atone for the agony, and some experiments which would have cost too dearly had not your art furnished the aid to science, and the anodyne and anæsthetic to the patient. What a humane and glorious science is that which like Christ give articulateness to speech, and out of the mouths of babes and sucklings ordains praise. What a public benefactor is every dentist, who, in the skillful exercise of his profession, gives a relish to the food we eat, an aroma to the air we breathe; and dissipates the clouds of misanthropic discontent, with the genial light of a benign and generous philanthropy. And here it is well to vindicate your profession from a popular and ignorant idea that it is not one of the learned branches of medical education. Some people suppose any one may readily be a dentist, without much reading, experience or practice. But the fact is, (as we see,) it embraces one of the most important parts of surgery, it involves a knowledge of Physiology, Anatomy, Pathology, Therapeutics, Medicine, Chemistry and Metallurgy. The reason why the credulous and ignorant have formed such false opinions, is because such an amount of presumptuous quackery prevails as to justify almost any superstition. And we have heard of an ignorant practitioner, who being baffled in his effort to extract a deeply rooted tooth, tied his patient to a fixed point and fired a blank cartridge to make him draw back and do in his fright, what the so-called dentist could not do with his hands. And I doubt not you have seen this depreciation of your profession in the complaints made of charges for well-earned skill. "You charge me two dollars, said an ignorant patient for extracting a tooth in a *moment*, when a friend of mine only paid fifty cents, and he was drawn all around the room." The science which does without pain, and in a moment the work which cost hours to the bungling doctor and agonies to the poor patient, deserves a better judgment and a higher appreciation from an enlightened public.

There is a suggestion, which, I trust it will not weary you if I dwell upon it, and that is *the temptation you are under to disregard general laws.* The natural tendency of the mind, is to observe facts separately—to isolate phenomena. The empiricism which so widely prevails, is owing to this tendency of the popular mind to seize exclusively upon facts; in other words, it is because general principles are ignored, because a large generalization is neglected, a nice perception of cause and effect is overlooked, and truth is sought for in single examples. All true science has constant reference to the causal principle. The law which determines any phenomenon, is the key to unlock that department of nature to which it belongs. The only true method in any inductive process of reasoning, is from effect to cause. And as it takes more than one swallow to make a summer, so it demands more than one fact to establish a law in any science. The tendency to be more impressed by affirmative than with negative facts, was noticed by Lord Bacon, as a source of great error in the study of the sciences. It is impossible to construct a harmonious and true system upon any single one, or even a series of phenomena. Logic teaches us that it is fallacious to draw a universal conclusion from a particular premise. The wider the field of induction, the more abundant will be the harvest of real and precious truth. The true progress of your scientific profession must depend upon generalization—upon the classification of phenomena under established law. Hence, the great advantage to be derived from the practice of reporting and publishing cases in medical journals is, that while they furnish a vast array of facts, at the same time they contribute to the induction of the general laws of pathology. And it was owing to a neglect of this important principle, that for a time, the impositions of the Royal Cure, the Sympathetic Powder, and Metallic Tractor, so widely prevailed.

Again let me remind you, in your devotion to your profession and its kindred studies, *to guard against the temptations of materialism.* The attractions of seemingly learned science, which would arraign the testimony of revealed truth, and claim a wisdom above that of the Bible, have lured many into deadly error. There is a true philosophy in all science, but any system which seeks to account for the existence of the universe and of man, the crowning work of God, upon any other principle than that of the Bible must be false. And materialism virtually does this. It would teach us that life is the result of chemical forces; that man is but the last of a series of progressive developments which began with the first nucleated cell. Such a theory would rob our nature of its highest attributes. It does not account to us for conscience, nor explain the distinctions and origin of our sense of right and wrong; of virtue and vice. How can materialism explain the vast disparity in the intellectual powers of man. Are they dependent solely upon brain tissue? Are the immortal poems of Homer and the lofty meditations of Plato, the result of physiological condition? As the particles of matter which compose our bodies are constantly undergoing new forms, can materialism tell us by what law it is that memory retains or transmits knowledge, or that reason deduces inferences from premises dependent upon elements long since disorganized. As well consent that the wires of the telegraph are the instruments of thought as to maintain that mind and matter are one. They are intimately united! As Bishop Butler, in his Analogy, states: "They mutually affect one another;" but they are not identical or essentially co existent and interdependent. No study convinces us more profoundly of the presence and power of the great Creator than that of the mechanical adaptations of our physical organism to the uses of the indwelling spirit.

The ear may be said to be a kind of stethoscope by which we learn to note the breathing of animated nature and to sound the great lung system of the atmosphere. The eye combines in the highest perfection the properties both of the microscope and the telescope. The nerve-system is a sort of telephone, interpreting to us the voices of nature which awaken the responses of the soul. Yet intimate as the sympathies of mind and matter are we must distinguish them. How many examples there are of the superiority and independence of the intellect to the body. Cicero, Milton and Burke, even in the decline of their life, gave proof that their immortal thoughts were still fresh and brilliant, and that the lamp of their genius was undimmed by the earthen vase; and as it dissolved, the flashes shone forth more distinctly than ever.

Of all forms of material existence, we may say, with the Psalmist, "They shall perish and they shall wax old as doth a garment, and as a vesture shalt thou fold them up, and they shall be changed." But to the soul of man it were no irreverence to accommodate a passage of holy writ, which so truly expresses its nature: "But thou remainest and thy years shall not fail."

Materialism regards all science as an experiment, and accounts for all phenomena by what it calls "the laws of nature." But nothing can satisfy the demands of our reason and the yearnings of our hearts, but the existence and confessed presence and power of the Personal God.

Well has the poet Cowper expressed it:—

"Some say that in the origin of things,
When all creation started into birth,
The infant elements received a law
From which they swerve not since."

"That under force
Of that controlling ordinance they move,
And need not his immediate hand who first,
Prescribed their course to regulate it now.
But how should matter occupy a charge
Dull as it is, and satisfy a law
So vast in its demands, unless impelled
To ceaseless service by a ceaseless force,
And under pressure of some conscious cause?
He feeds the secret fire,
By which the mighty process is maintained.
Who sleeps not—is not weary; in whose sight
Slow circling ages are as transient days;
Whose work is without labor,
Whose designs
No flaw deforms, no difficulty thwarts
And whose beneficence no charge exhausts

To believe that law, "*a vis formitiva*," is the great first cause of all effect, would be as unreasonable as to believe that a piece of mechanism could be constructed and moved by itself. Even if we were to grant that all the laws of nature were made to work without the superintending agency of Jehovah, and that this complex machinery was like Babbage's Computing Machine, to work out its own results; a great clock-work, wound up once, and then left to control the oscillations of human experience, we could not avoid pre-supposing a designing mind as the originator of it all. The material world is a vast cathedral, in which the chorus of his praises is perpetually hymned.

All philosophy has failed to give any satisfactory explanation of human existence. Atkinson's teachings of the nature and development of man, are not one step in advance of the heathen Lucretius. The ancient epicureans considered the soul corporeal, but of a finer substance than the body. The Stoics thought it was ignited air, and Pythagoras that it was harmony. Yet they all knew really as much of the cause of life and the nature of the soul as the latest authorities of materialism. They, however, confessed their ignorance. They felt the need of a light above that of earth-born philosophy. Aristotle exclaimed, "cause of causes pity me;" and yet these prophets of materialism spurn prayer, and ridicule the doctrine of a providence.

Never does a student of science, and most of all of medical science, which appeals to the best sympathies and touches the finest cords of our nature, enter into a just appreciation of his responsibilities to society, until he has learned to view his studies and investigations in the light of Christianity. How it ennobles every pursuit to realize that every truth is a link in the chain which binds us to God! What great names, like stars of the first magnitude, flush the firmanent with the lustre of their devotion to Revealed Truth. Bacon said: "I have found thee in the garden and the fields, but most of all in thy temple." We see, among physicians, Gregory and Boerhave and Sydenham and Rush, who like the Magi, have laid their learning and influence and wealth at the feet of the great teacher, as votive offerings to truth and holiness. Who are the men who have gladdened life the most by their example and counsels, who have adorned the highest positions by wisdom, moderation and integrity; who have contributed the most solid and precious treasure to learning, and left their names as a heritage of blessing to their fellows? They are the men who have learned to recognize a higher teaching than that of the retort and the crucible only, and who have walked with reverent steps and uncovered brow the great temple of natural science.

They have felt the truth and beauty of Cowper's lines:

> "Thou giv'st its lustre to an insect's wing,
> And wheel'st thy throne upon the rolling worlds;
> From thee is all that cheers the life of man.
> His high endeavor, and his glad success,
> His power to suffer, and his will to serve.
> But oh! thou bounteous giver of all good,
> Thou art, of all thy gifts, thyself the crown,
> Give what thou canst; without thee we are poor,
> And with thee rich, take what thou wilt away."

Shall I remind you of the oft repeated fact that your education is just begun. There are many temptations to content ourselves with the present achievements. Your diplomas are a pledge of your loyalty to conscientious study. The motto of Michael Angelo was "I still learn." The constant advances in your profession will compel you to be men of reading and inquiry. The intense activities of the age have quickened the intellectual energies of men of every profession. It requires no ordinary effort to keep pace with the progress of learning. He who relaxes his energies or looks back, must soon be left a laggard in the race, and suffer the oblivion and defeat which are the doom of indolence. Let the same spirit which prompted the motto of a lover of his art, be yours, "*Nulla dies sine linea.*" The aids which are now furnished are an additional incentive to close and constant study. The nicest inventions in mechanical instruments; the discoveries in chemistry; the knowledge of the affinity which metals possess for the non-metallic, and the investigation of their properties, and indeed the whole science of metallurgy have contributed to heighten the demands and the standard of your noble profession.

What has been done already, will inspire confidence in the capabilities and the promise of the future. The names of Galen and Fullopius, of Eustachius and Ambroise Taie, remind us of the masteries already achieved. We follow a long line of able and patient explorers, who, like John Hunter, of England, and Bichat, of France, prepared the way for Blake and Fox. We learn from the advertisements in newspapers of 1803, that the practice of making and cleansing teeth appears to have been in the hands of silversmiths or jewellers. It was to a Baltimorean that Dental Surgery owes its first claims to rank as a distinct branch of science. In 1826 the principles of Dental Surgery, by Leonard Koecker, M. D., who practised his profession for fifteen years here, and in Philadelphia, appeared in London. And since then every year has added to the glory and success of the profession.

Baltimore has the credit of publishing as early as 1839, the *American Journal and Library of Dental Science.* And we see this College, with its rich equipments and its skilled professors, the monument and the witness of the advances in this humane and scientific department of knowledge.

The very city, then, with its historic renown appeals to you to "quit you like men." How superior are your advantages; what fresh discoveries; what new light floods your path; what great names appeal to you to be men of patient, earnest, diligent study. We have great reason to be proud of our city to-day.

It is not only the city of monuments, with the memories carved in stone of the heroes of North Point, and with the noble image of the soldier and statesman who was "first in peace and first in war and first in the hearts of his countrymen;" but it is the city of schools and colleges and universities. Its libraries and lecture halls incite the dullest aspirations and feed the purest tastes. Its churches and asylums remind us, that while states may fall and arts may fade, yet there abide these virtues which never die, Faith, Hope and Charity.

